# Retained rice cake in the stomach leading to potential intestinal obstruction

**DOI:** 10.1002/jgf2.548

**Published:** 2022-04-19

**Authors:** Akihiro Saitsu, Norihiro Kojima, Kotaro Kunitomo

**Affiliations:** ^1^ Department of General Medicine Taragi Municipal Hospital Kumamoto Japan; ^2^ Department of General Medicine Kumamoto Medical Center Kumamoto Japan

## Abstract

A 66‐year‐old Japanese woman presented with upper abdominal pain and nausea after having eaten several rice cakes. An abdominal CT scan showed retained rice cakes in the stomach, which led to intestinal obstruction three days later.

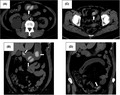

A 66‐year‐old Japanese woman who had a thyroidectomy for thyroid carcinoma presented at our Department of Gastroenterology with upper abdominal pain and nausea, which she had been experiencing for the past 6 h. She had eaten several rice cakes without sufficiently chewing them the day before. Her vital signs were within normal limits. A physical examination revealed mild tenderness of the upper abdomen. An abdominal CT scan showed multiple high‐density bodies in the stomach, the largest of which was an oval ball‐like shaped body measuring 27 mm × 30 mm; however, no intestinal obstruction was visible (Figure 1A,B). Three days later, she was re‐examined with no improvement in her symptoms. An abdominal CT scan showed high‐density bodies in the ileum and the dilated ileum proximal to obstruction (Figure 1C,D), indicating intestinal obstruction. She was treated with fasting and parenteral intravenous saline, and she fully recovered within 48 h.

**FIGURE 1:**
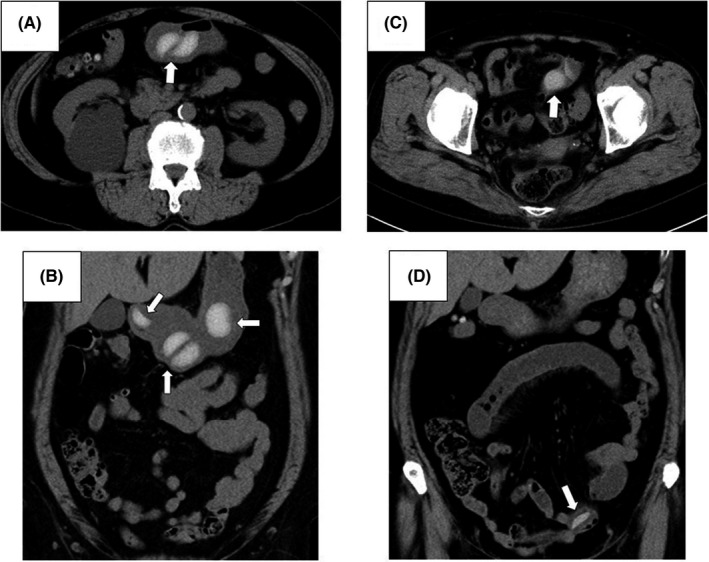
(A) Axial section of abdominal plain CT scan shows (arrow) high‐density intraluminal ingested food item (rice cakes) in stomach. (B) Coronal reformation of abdominal plain CT scan shows (arrow) high‐density bodies (rice cakes) in stomach. (C) Axial section of abdominal plain CT scan shows (arrow) high‐density body (rice cake) in ileum. (D) Coronal reformation of abdominal plain CT scan shows (arrow) obstructing high‐density body (rice cake) in ileum with dilation of ileum proximal to obstruction

Since rice cake is a traditional Japanese food and is becoming popular worldwide, intestinal obstruction caused by rice cakes may be encountered more frequently in regions outside Japan as well. In earlier studies, rice cake as a foreign body (retained rice cake) in the stomach was found to be rare, but if the diameters of pieces of retained rice cake are ≥20 mm, they may remain and result in intestinal obstruction.^1^ Therefore, if larger rice cake pieces are found in the stomach, they should be monitored carefully in case of intestinal obstruction, which, if occurs, should be managed appropriately.

## CONFLICT OF INTEREST

The authors have stated explicitly that there are no conflicts of interest in connection with this article.

## INFORMED CONSENT

The patient provided informed consent for the publication of this report.
